# Evaluation of factors related to organic food consumption and orthorexia nervosa tendencies

**DOI:** 10.1002/fsn3.4029

**Published:** 2024-02-16

**Authors:** Osman Bozkurt, Betul Kocaadam‐Bozkurt

**Affiliations:** ^1^ Department of Nutrition and Dietetics, Faculty of Health Sciences Erzurum Technical University Erzurum Turkey

**Keywords:** awareness, ecological footprint, obesity, organic food consumption, orthorexia nervosa

## Abstract

The relationship between organic food consumption, awareness of reducing ecological footprint, and orthorexia nervosa (ON) tendencies has yet to be explored in detail. This study aimed to determine factors related to organic food consumption and ON tendencies in young adults. Also, the relationship between organic food consumption, awareness of reducing ecological footprint, and ON tendencies was investigated. This study was conducted with 887 young adults (58.4% female, 41.6% male, aged 18–25 years, mean age 20.8 ± 2.55 years). Study data were obtained with a web‐based questionnaire. The questionnaire included socio‐demographic characteristics, anthropometric measurements, the Organic Food Consumption Scale (OFC), ORTO‐11, and the Awareness Scale for Reducing Ecological Footprint (ASREF). While 17.2% were overweight or obese, 74.9% had a normal body weight. There were no differences by sex in terms of ORTO‐11, OFC, and ASREF total scores (*p* > .05). Significant positive correlations were found between ASREF and OFC (*p* < .001), while negative correlations were found for ORTO‐11 and OFC (*p* < .001). A higher ASREF and increased ON tendencies predicted increased organic food consumption (*p* < .05). Furthermore, higher organic food consumption behaviors and awareness of reducing ecological footprint predicted increased ON tendencies (*p* < .05). Findings from this study will contribute to a deeper understanding of the factors associated with organic food consumption and ON tendencies among individuals. By shedding light on the interplay between ecological awareness, organic food consumption, and orthorexic tendencies, policymakers and health professionals can develop targeted interventions to promote sustainable and healthy food consumption.

## Graphical abstract

A higher awareness scale for reducing the ecological footprint (ASREF) and increased orthorexia nervosa (ON) tendencies were related increased organic food consumption (OFC). Higher OFC and ASREF scores were related increased ON tendencies. ASREF, ORTO‐11, and OFC total scores did not differ by sex. BMI was not related with OFC total scores and ON tendencies.

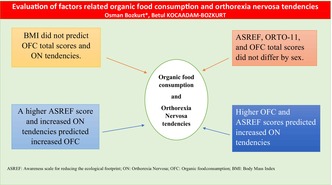

## 1 INTRODUCTION

The environment is subjected to greater stress due to increasing food demand. It is necessary to take important measures to reduce irreversible damage to the environment and climate change (Edenhofer, 2014). One of the measures is that dietary habits must be changed for the human population to maintain its current lifestyle. In that context, the Food and Agriculture Organization (FAO) defined sustainable diets as “with low environmental impacts which contribute to food and nutrition security and to healthy life for present and future generations. They are protective and respectful of biodiversity and ecosystems, culturally acceptable, accessible, economically fair and affordable; nutritionally adequate, safe and healthy, while optimizing natural and human resources” (FAO, 2010). Sustainable environmental awareness is essential to changing consumer eating behaviors. Thus, with the adoption of sustainable diets, a lifestyle that causes less harm to the environment can be created (Goldstein et al., 2017).

The increasing severity of environmental issues has prompted the emergence of novel concepts aimed at fostering awareness. The concept of “ecological footprint” is one of them. Using a new calculation method for measuring the productivity and quantity of natural resources, consuming natural resources, and devising solutions to prevent damage to these concepts, the purpose of this concept is to determine which factors cause the damage (Tekindal et al., 2021). Recent research has found that higher awareness of reducing ecological footprint is associated with higher sustainable and healthy eating behaviors (Kocaadam‐Bozkurt & Bozkurt, 2023).

It is known that 25% of greenhouse gas emissions stem from food production (Edenhofer, 2014). The most critical factor in forming this situation is fossil fuels and carbon/water footprints while transporting food products (Goldstein et al., 2017). Within the scope of the Paris Agreement, it aims not only to reduce fossil fuel consumption but also to reduce greenhouse gas emissions in agriculture significantly (Bryngelsson et al., 2016). For this purpose, agriculture and nutrition models with a low ecological footprint and that protect the ecosystem and biodiversity should be implemented (Dominati et al., 2019). According to studies, organic foods are preferred due to their low ecological footprint (Hansen et al., 2018; Limnios et al., 2016). Organic farming has an environmentally friendly, low ecological footprint, and no use of chemical pesticides and fertilizers (Treu et al., 2017). Due to the increased demand for organic food products in recent years, organic agriculture has developed in many countries (Ditlevsen et al., 2019; Treu et al., 2017). This demand for organic food products is believed to be sustainable, have a low environmental impact, and the public prefers to consume healthy foods (Hansen et al., 2018). Studies have found that the desire for healthy and environmentally friendly nutrition is one reason for organic food preference (Denver & Christensen, 2015; Huber et al., 2011). Also, studies have shown a positive relationship between a healthy lifestyle and a preference for organic food (Pelletier et al., 2013).

Adopting a healthy lifestyle can be achieved with adequate and balanced nutrition (Voglino et al., 2021). However, this may become an unexpected outcome because of the restriction of certain foods and obsession with some foods for healthy eating (Costa et al., 2017). In the definition of orthorexia nervosa (ON), to be obsessed with healthy eating or think about healthy eating in a pathological dimension, along with malnutrition and loss of body weight, psychosocial damages can be seen (Cena et al., 2019). Orthorexics are more concerned about the quality and source of food than its quantity. At the same time, these individuals may be obsessed with nutrition, such as preparing healthy foods and not choosing foods they consider unhealthy or unnatural. When purchasing food, they take time to examine the origin, production method, package details, and labels (Duarte et al., 2019). Also, they avoid processed, fatty, and salty foods, while paying attention to organic nutrition (Hanganu‐Bresch, 2020). A study stated that organic foods are the correct healthy food choices according to orthorexic individuals (Cena et al., 2019). Also, there are studies on the increase in organic food consumption of orthorexic individuals due to their perceived health benefits (Baydas, 2021; Duarte et al., 2019; Voglino et al., 2021). In this regard, those who prefer organic food may be at risk of ON tendencies.

As research investigating the relationship between organic food consumption, awareness of reducing ecological footprint, and ON tendencies has yet to be explored in detail, the present study will make a significant contribution to the literature. First, this study determines the predictors of ON tendencies among young adults. Second, the variables predicting organic food consumption are detected among young adults. Finally, the relationship between organic food consumption, awareness of reducing ecological footprint, and ON tendencies is investigated.

## 2 METHODS

This cross‐sectional study was conducted with 887 young adults (58.4% female, 41.6% male, aged 18–25 years, mean age 20.8 ± 2.55 years) between November 2022 and May 2023 in Erzurum/Turkiye (one of the cities in the east of Turkiye). Study data were obtained with a web‐based questionnaire via Google Forms. Participants were selected by the snowball sampling method who lived in Erzurum city. Inclusion criteria were meeting the age criteria (18–25 years), not having chronic health conditions, psychological disorders, or eating disorders, consenting to participate, and not following a special diet or eating model. Exclusion criteria were being pregnant or breastfeeding, not meeting the age requirement, and following a special diet or eating model. Ethical permission was obtained from the Erzurum Technical University Ethics Committee (Meeting Number: 11; Decision Number: 20; Date: 17.11.2022). The study was carried out following the principles outlined in the Helsinki Declaration. Informed consent was obtained from the participants.

The questionnaire included socio‐demographic characteristics, anthropometric measurements, the Organic Food Consumption Scale (OFC), ORTO‐11, and the Awareness Scale for Reducing Ecological Footprint (ASREF).

### 2.1 Organic food consumption scale

Eti İçli et al. (2019) developed and validated the Organic Food Consumption Scale (OFC), a novel tool for measuring organic food consumption in Turkish adults (Eti İçli et al., 2019). The OFC scale consists of 18 items and six subscales. All items were measured on a five‐point Likert‐type scale (1: strongly disagree, 3: neutral, and 5: strongly agree) (Eti İçli et al., 2019). The subscale norms relate to the thoughts of consumers' friends or peers regarding organic food purchasing tendencies. The health consciousness subscale relates to the care and attention that consumers show to their health. The self‐identity subscale relates to the perceptions of organic food consumption as to whether it is environmentally friendly and whether it is healthy food. The benefits of Consuming Organic Food subscale relates to consumers finding organic food consumption useful, meaningful, and good. The positive Moral Attitudes subscale relates to the thoughts of consumers about doing something good for society and their families through the consumption of organic food. The Information Seeking subscale refers to the level of knowledge of consumers regarding the consumption of organic food products. Low scores on the scale indicate decreasing tendencies for organic food consumption, while high scores show increasing tendencies for organic food consumption.

### 2.2 ORTO‐11 test

The ORTO‐15 is a 15‐item self‐report instrument developed by Donini et al to evaluate orthorexic tendencies (Donini et al., 2005). The items assess an individual's selection, purchase, preparation, and consumption of healthy foods. Each item is rated on a four‐point Likert scale ranging from “always” to “never.” In the Turkish version's validity and reliability study, Arusoğlu et al. offered to exclude four items; the scale became known as ORTO‐11 (Arusoğlu et al., 2008). Each item is scored in a 4‐point Likert‐type from “always” to “never.” Low scores indicate higher orthorexic tendencies (Arusoğlu et al., 2008).

### 2.3 Awareness scale for reducing ecological footprint

Tekindal et al. (2021) developed the ASREF in Turkish adults as a measurement tool that will raise awareness of damage reduction by highlighting the concept of environmental responsibility in the light of increasing ecological problems. The scale consists of six subscales: Energy, Under the Laws, Transportation, Recycling, Food, and Water Consumption. The total score is obtained by summing the subscales and scoring using the 5‐point Likert scale (strongly disagree to strongly agree, 1 to 5 points). As the total and subscale scores increase, awareness of reducing the ecological footprint increases (Tekindal et al., 2021).

### 2.4 Anthropometric measurements

Height and body weight measurements were taken based on the self‐reports of individuals. By dividing the body weight by the square of the height, the body mass index (BMI) was calculated. Body mass index below 18.50 kg/m^2^ was classified as underweight, between 18.50–24.99 kg/m^2^ as normal, 25.0–29.99 kg/m^2^ as overweight, and above 30.0 kg/m^2^ as obese (“World Health Organisation. (2006). Global database on Body Mass Index: BMI Classification.Geneva: World Health Organization.”).

### 2.5 Statistical analysis

The data were analyzed using SPSS Statistics for Windows (IBM SPSS Statistics for Windows, Version 23.0; Armonk, NY: IBM Corp). Normality testing was performed to determine whether the parametric test assumptions were met. Mean (*ᵡ¯*) and standard deviation (SD) values were used for continuous variables, and categorical variables were presented as frequency (*n*) and percentage (%). The Chi‐square test was used for categorical variables, the Student's *t*‐test or ANOVA test was used for normally distributed variables, and the Mann–Whitney U test or Kruskal–Wallis test was used for non‐normally distributed variables in comparisons between groups. According to the data distribution, a Spearman or Pearson correlation coefficient was conducted to analyze the correlation between the parameters. A multiple linear regression model was used to identify independent predictors of organic food consumption and ON tendencies.

## 3 RESULTS

The general characteristics of the individuals participating in the study are given in Table 1. The mean age of the individuals was 20.9 ± 2.55 (41.6% men, 58.4% women). While 17.2% were overweight or obese, 74.9% had a normal body weight. 46.3% of participants were orthorexic, according to ORTO‐11. The total OFC and ASREF scores were 59.6 ± 10.01 and 3.7 ± 0.54, respectively. There were no differences by sex in terms of ORTO‐11, OFC, and ASREF total scores (*p* > .05) (data not shown).

**TABLE 1:** General characteristics of the participants (*n*: 887).

	*n*	%
Sex		
Men	369	41.6
Women	518	58.4
BMI classification		
Underweight	70	7.9
Normal	664	74.9
Overweight	112	12.6
Obese	41	4.6
	**X¯±SD**	
Age (years)	20.9 ± 2.55	
ORTO‐11 total score	29.4 ± 4.10	
OFC scale		
Total score	59.6 ± 10.01	
Norms	11.0 ± 2.80	
Health consciousness	10.5 ± 2.23	
Self‐Identity	9.5 ± 2.39	
Benefits of consuming organic food	15.0 ± 2.95	
Positive moral attitudes	7.1 ± 1.75	
Information‐seeking	6.8 ± 1.68	
ASREF		
Total score	3.7 ± 0.54	
Energy	3.9 ± 0.66	
Under the Laws	4.2 ± 0.65	
Recycling	3.6 ± 0.63	
Transportation	3.5 ± 0.78	
Food	3.6 ± 0.60	
Water Consumption	3.6 ± 0.69	

Abbreviations: ASREF, Awareness Scale for Reducing Ecological Footprint; BMI, Body Mass Index; OFC, Organic Food Consumption.

Table 2 shows the variables (BMI, ORTO‐11, and ASREF) across the quartile categories of the OFC. There was a significant difference among quartiles for all variables (<.001). While BMI and ORTO‐11 scores were the highest in Q1, ASREF total score and subscale scores were the highest in Q4 (*p* < .001). Significant positive correlations were found between ASREF and OFC (*p* < .001), while negative correlations were found for ORTO‐11 and OFC (*p* < .001) (Table 3).

**TABLE 2:** Evaluation of variables according to OFC quartiles.

	OFC quartiles				*p*
	Q1 (*n*: 222) (18.0–54.0)	Q2 (*n*: 240) (55.0–61.0)	Q3 (*n*: 213) (62.0–66.0)	Q4 (*n*: 212) (67.0–82.0)	
BMI (kg/m^2^)	23.4 ± 5.26^a^	22.2 ± 4.27^b^	21.8 ± 2.94^b^	22.7 ± 3.18^a,b^	<.001
ORTO‐11 scores	32.1 ± 3.71^a^	29.9 ± 3.29^b^	27.9 ± 3.51^c^	27.4 ± 4.13^c^	<.001
Awareness scale for reducing ecological footprint					
Total score	3.3 ± 0.56^a^	3.6 ± 0.45^b^	3.9 ± 0.36^c^	4.1 ± 0.41^d^	<.001
Energy	3.5 ± 0.81^a^	3.8 ± 0.55^b^	4.0 ± 0.54^c^	4.2 ± 0.52^d^	<.001
Under the laws	3.9 ± 0.80^a^	4.2 ± 0.61^b^	4.3 ± 0.53^c^	4.4 ± 0.49^c^	<.001
Recycling	3.3 ± 0.71^a^	3.5 ± 0.54^b^	3.7 ± 0.57^c^	3.9 ± 0.54^d^	<.001
Transportation	3.2 ± 0.81^a^	3.4 ± 0.74^a^	3.7 ± 0.67^b^	3.9 ± 0.69^b^	<.001
Food	3.2 ± 0.55^a^	3.6 ± 0.50^a^	3.8 ± 0.49^b^	3.9 ± 0.58^b^	<.001
Water consumption	3.1 ± 0.70^a^	3.4 ± 0.64^a^	3.8 ± 0.56^b^	3.9 ± 0.55^b^	<.001

*Note*: a,b,c,d: The groups with the same letters within a row are not significantly different according to pairwise comparisons.

Abbreviations: ASREF, Awareness Scale for Reducing Ecological Footprint; BMI, Body Mass Index; OFC, Organic Food Consumption.

**TABLE 3:** Evaluation of the relationship between OFC, ORTO‐11, and ASREF (correlation coefficient = *r*).

	ORTO‐11 total score	ASREF total score
OFC scale		
Total score	−0.473**	0.564**
Norms	−0.219**	0.286**
Health consciousness	−0.331**	0.393**
Self‐Identity	−0.363**	0.341**
Benefits of consuming organic food	−0.348**	0.373**
Positive moral attitudes	−0.378**	0.375**
Information‐seeking	−0.312**	0.426**

*Note*: Spearman or Pearson correlation coefficient (*r*), ^**^ *p* < .001.

Abbreviations: ASREF, Awareness Scale for Reducing Ecological Footprint; OFC, Organic Food Consumption.

When the factors (BMI, ASREF, and OFC total scores) related to ORTO‐11 total scores were evaluated with multiple linear regression analysis, the model was significant (*R* ^2^: 0.231, *p* < .001). ON was related to the ASREF and OFC total scores (*p*< .05) (Table 4). Higher organic food consumption behaviors and ASREF predicted increased ON tendencies. When the factors (BMI, ASREF, and ORTO‐11 total scores) related to OFC total scores were evaluated with multiple linear regression analysis, the model was significant (*R* ^2^: 0.422, *p* < .001). OFC total score was related to ORTO‐11 and ASREF total scores; however, BMI was unrelated (*p* > .05) (Table 5). A higher ASREF and increased ON tendencies predicted increased organic food consumption.

**TABLE 4:** Multiple linear regression analysis for ON tendencies prediction^a^.

Model		ORTO‐11 total score					
	*B*	SE	*ß*	*t*	*p*	Lower bound	Upper bound
Constant	41.677	1.361		30.613	**<.001**	39.005	44.349
BMI (kg/m^2^)	0.032	0.031	.032	1.036	.300	−0.029	0.093
ASREF total score	−0.379	0.280	−.050	−1.177	**.047**	−0.929	0.171
OFC total score	−0.182	0.015	−.443	−0.443	**<.001**	−0.210	−0.153
				***R*** ^**2**^ **: .231 *p* < .001**			

The bold values indicate significance at *p* < 0.05.

Abbreviations: ASREF, Awareness Scale for Reducing Ecological Footprint; BMI, Body Mass Index; OFC, Organic Food Consumption; ON, orthorexia nervosa.

^a^ Adjusted for sex.

**TABLE 5:** Multiple linear regression analysis for organic food consumption prediction^a^.

Model		OFC total score					
	*B*	SE	*ß*	*t*	*p*	Lower bound	Upper bound
Constant	49.852	3.779		13.193	**<.001**	42.436	57.269
BMI (kg/m^2^)	0.122	0.066	.050	1.858	.064	−0.007	0.251
ASREF total score	8.813	0.514	.476	17.155	**<.001**	7804	9821
ORTO‐11 total score	−0.812	0.066	−.333	−12.356	**<.001**	−0.941	−0.683
				***R*** ^**2**^ **: .422 *p* < .001**			

The bold values indicate significance at *p* < 0.05.

Abbreviations: ASREF, Awareness Scale for Reducing Ecological Footprint; BMI, Body Mass Index; OFC, Organic Food Consumption.

^a^ Adjusted for sex.

## 4 DISCUSSION

In our study, there were no differences by sex in terms of ORTO‐11, OFC, and ASREF total scores. BMI and ORTO‐11 scores were the highest in Q1, according to OFC total scores; ASREF total score and subscale scores were the highest in Q4. Significant positive correlations were found between ASREF and OFC, while negative correlations were found for ORTO‐11 and OFC total scores. In regression analysis, the OFC total score was related to the ORTO‐11 and ASREF total scores; however, BMI was unrelated. Higher ASREF and increased ON tendencies predicted increased organic food consumption. ON was related to the ASREF and OFC total scores. Higher organic food consumption behaviors and ASREF predicted increased ON tendencies.

### 4.1 Evaluation of the factors related to ON tendencies

Most studies that investigated ON prevalence were conducted with university students and found a high ON prevalence (~50%–70%) (Abdullah et al., 2020; Fidan et al., 2010; Oberle et al., 2017; Sanlier et al., 2016). In addition, studies conducted in recent years have observed an increased prevalence of ON in young adults (Skella et al., 2022). The ORTO‐11 scale is a scale without cut‐off values for the diagnosis of ON. Low scores indicate a tendency toward ON (Arusoğlu et al., 2008). Therefore, the ON prevalence of our sample could not be calculated. However, peer pressure and social media pressure may be associated with an unrealistic body image, a desire for thinness, and various unhealthy weight‐control methods (Bazzano, 2006). Also, there is much information pollution online about healthy eating (McComb & Mills, 2019; Turner & Lefevre, 2017).

Sex and BMI are two demographic variables repeatedly linked to ON (Oberle et al., 2017) and should therefore be considered in the present study. Studies evaluating the relationship between sex, BMI, and ON show different results. In some studies, BMI and sex are significant risk factors for ON (Abdullah et al., 2020; Oberle et al., 2017), but they are not related in other studies (Plichta et al., 2019; Sanlier et al., 2016). In our study, ORTO‐11 total scores did not differ by sex. Furthermore, BMI was not found to be a risk factor for ON tendencies. This may be because most of the study population (74.9%) was within the normal BMI range.

One of the critical findings in our research was that the increase in ASREF and OFC scores was a risk factor for ON tendencies. People with ON may develop unhealthy dietary restrictions due to their preoccupation with maintaining a healthy, balanced diet (Costa et al., 2017). The emphasis on reducing ecological footprints encourages reducing carbon footprints, safeguarding biodiversity, ethical agricultural practices, and sustainable and mindful eating (Kocaadam‐Bozkurt & Bozkurt, 2023). Although awareness of reducing the ecological footprint and organic food consumption is carried out to improve and protect the health and environment, having excessive thoughts about food is among the undesirable behaviors. This can cause people to adopt strict dietary rules and eliminate certain food groups, such as processed foods and those with a higher carbon footprint (animal products). Studies evaluating the effects of ecological footprint awareness and organic food consumption trends on ON are limited. Furthermore, to the best of our knowledge, there is no study evaluating the effect of awareness of reducing ecological footprints on the risk of ON tendencies. According to our results, such behaviors may act as risk factors for ON tendencies. In a study conducted in Italy, potential organic store customers were assessed for ON risk. The study showed an association between organic food consumption and ON symptoms (Voglino et al., 2021). Similarly, it was shown that people with higher orthorexic tendencies are more likely to consume organic and healthy food (Plichta et al., 2019). Individuals at risk for ON may perceive organic and sustainable foods as innately healthier and view their consumption as an option for achieving optimal health and well‐being. However, this can result in rigid thinking, an excessive preoccupation with the quality of food, and a narrowing of food choices. Preventing ON needs a balanced strategy that considers food choices' environmental and psychological aspects. Encouraging individuals to focus on well‐being, mindful eating, body positivity, and the importance of an adequate and balanced diet rather than rigid dietary rules can help reduce the risk of ON while promoting sustainable and organic food consumption. In order to prevent unexpected health outcomes, it is critical to obtain accurate information about healthy and sustainable nutrition from health professionals.

### 4.2 Evaluation of the factors related to organic food consumption

It is stated that while the choice of organic food is considered feminine by male consumers, this is not the case for women. Moreover, there is a perception that women prefer healthy foods and that men prefer unhealthy foods (Shin & Mattila, 2019). Some studies support this situation, and it has been found that women consume more organic food than men (Akhondan et al., 2015; Martins et al., 2019). On the other hand, it was also stated that the awareness of organic food or organic food purchasing tendencies did not change between the sexes (Fatha & Ayoubi, 2021; Gundala et al., 2022). In this study, the effect of sex on organic food consumption was not found. The difference in the results of these studies may be due to the differences in the age groups of the samples.

It has been reported that there may be a difference between generations related to organic food consumption. In a study, Z‐generation consumers tend to consume less organic food than other generations (Özden & Yapici, 2021). According to studies on organic food consumption, the X and Y generations consume more organic food (Davies et al., 1995), while the Y and Z generations are more conscious of organic food products (Akın et al., 2010). Our study did not evaluate the difference in organic food consumption between generations because most participants were from the Z generation. Future research can evaluate generational differences in organic food consumption trends.

The exposure to pesticides, antibiotics, and hormones in conventional food production is significantly higher than that in organic foods, and these compounds can increase BMI, abdominal obesity, and insulin resistance (Heindel et al., 2017). A meta‐analysis study shows that consumption of organic foods is associated with a lower risk of obesity (Bhagavathula et al., 2022). In a cohort study conducted in France, participants with a high frequency of organic food consumption had a lower risk of obesity (Kesse‐Guyot et al., 2017). In another study, organic food consumption was negatively associated with both BMI and obesity (Gosling et al., 2021). In our study, while BMI was significantly higher in Q1 according to OFC quartiles, BMI had no effect in regression analysis. This may be because most of the study population (74.9%) was within the normal BMI range. Future research can evaluate the relationship between obesity and organic food consumption.

Many studies have emphasized the significance of reducing the ecological footprint for achieving sustainability goals (Amprazis et al., 2023; Long et al., 2020). Individuals who are conscious of their ecological footprint may be more inclined to adopt sustainable consumption practices, such as choosing organic foods. (Limnios et al., 2016; Özden & Yapici, 2021) Some studies have found a positive relationship between environmental consciousness and organic food consumption (Ahmed et al., 2021; Wang et al., 2020). Similarly, Gundala and Singh (2021) found that adults who are more concerned about their health and the environment are more likely to consume organic foods (Gundala & Singh, 2021). In a study conducted in Turkey, the relationship between the ecological intelligence of consumers and their organic food consumption tendencies was evaluated, and the relationship between them was found to be statistically significant (Özden & Yapici, 2021). In a study conducted in France, organic consumers had healthier and more plant‐based foods than non‐organic consumers. Their diets were associated with a lower ecological footprint and reduced pesticide residue exposure (Kesse‐Guyot et al., 2022). Our study results support the idea that awareness of reducing ecological footprints increases the tendency to consume organic food.

One of the primary characteristics of ON is avoiding foods containing chemicals, artificial substances, or high amounts of fat, sodium, or sugar (Moroze et al., 2015). According to recent ON definitions, suitable food is primarily characterized as healthy/proper/correct, organic, and biologically uncontaminated (Cena et al., 2019). Some studies support the idea that orthorexic people choose organic food consumption (Duarte et al., 2019; Voglino et al., 2021). A similar result was shown in our study. Orthorexic tendencies may reinforce the preference for organic foods due to their perceived health benefits.

The study's strengths are as follows: large sample size; it is one of the first studies in which the relationship between organic food consumption, awareness of reducing ecological footprints, and ON was examined; and a study that states that increasing awareness of reducing the ecological footprint and organic food consumption scores may risk ON tendencies. The following limitations should be considered when evaluating study data. Firstly, the research is a cross‐sectional study conducted in Erzurum/Turkiye (one of the cities in the east of Turkiye) that was unable to establish a cause‐and‐effect relationship but was used to evaluate the relationship between the measured variables. Secondly, based on self‐reports, the participant's body mass index was calculated.

## 5 CONCLUSION

In conclusion, this paper provides comprehensive data on factors related to organic food consumption and ON tendencies. Increasing awareness of reducing the ecological footprints and orthorexic tendencies increased OFC scores in young adults. Also, the increase in ASREF and OFC scores may be risk factors for ON tendencies. There were significant associations between ON, organic food consumption, and awareness of reducing ecological footprints. Individuals at risk for ON tendencies may perceive organic and sustainable foods as innately healthier and view their consumption as an option for achieving optimal health and well‐being. Encouraging individuals to focus on well‐being, mindful eating, body positivity, and the importance of an adequate and balanced diet rather than rigid dietary rules can help reduce the risk of ON while promoting sustainable and organic food consumption.

Findings from this study will contribute to a deeper understanding of the factors associated with organic food consumption and ON tendencies among individuals. By shedding light on the interplay between ecological awareness, organic food consumption, and orthorexic tendencies, policymakers, and health professionals can develop targeted interventions to promote sustainable and healthy food consumption. Further research could explore the effect of sex and generational differences on organic food consumption and ON, as well as include sample groups with higher obesity prevalence.

## AUTHOR CONTRIBUTIONS

**Osman Bozkurt:** Conceptualization (lead); data curation (lead); formal analysis (lead); investigation (lead); methodology (lead); writing – original draft (lead); writing – review and editing (lead). **Betul Kocaadam‐Bozkurt:** Conceptualization (lead); data curation (lead); formal analysis (supporting); investigation (supporting); methodology (supporting); writing – original draft (supporting); writing – review and editing (lead).

## CONFLICT OF INTEREST STATEMENT

The authors have declared no conflicts of interest.

## ETHICAL APPROVAL

Ethical permission was obtained from the Erzurum Technical University Ethics Committee (Meeting Number:11; Decision Number:20; Date: 17.11.2022). The study was carried out in accordance with the principles outlined in the Helsinki Declaration. Informed consent was obtained from the participants.

## Data Availability

The datasets generated and/or analyzed throughout this study are publicly unavailable.
